# Clinical evaluation of viral acute respiratory tract infections in children presenting to the emergency department of a tertiary referral hospital in the Netherlands

**DOI:** 10.1186/s12887-014-0297-0

**Published:** 2014-12-10

**Authors:** Jairo Gooskens, Vishnu van der Ploeg, Ram N Sukhai, Ann CTM Vossen, Eric CJ Claas, Aloys CM Kroes

**Affiliations:** Department of Medical Microbiology, Leiden University Medical Center, Leiden, The Netherlands; Department of Pediatrics, Leiden University Medical Center, Leiden, The Netherlands

**Keywords:** Acute respiratory tract infection, Pediatric emergency department, Respiratory virus, Signs and symptoms, Clinical outcome

## Abstract

**Background:**

The relative incidence and clinical impact of individual respiratory viruses remains unclear among children presenting to the hospital emergency department with acute respiratory tract infection (ARTI).

**Methods:**

During two winter periods, respiratory virus real-time multiplex PCR results were evaluated from children (< 18 years) presenting to the emergency department of a tertiary referral hospital with ARTI that had been sampled within 48 hours of hospital presentation. In an attempt to identify virus-specific distinguishing clinical features, single virus infections were correlated with presenting signs and symptoms, clinical findings and outcomes using multivariate logistic regression.

**Results:**

In total, 274 children with ARTI were evaluated and most were aged < 3 years (236/274, 86%). PCR detected respiratory viruses in 224/274 (81.8%) children and included 162 (59%) single and 62 (23%) mixed virus infections. Respiratory syncytial virus (RSV) and human rhinovirus (HRV) single virus infections were common among children aged < 3 years, but proportional differences compared to older children were only significant for RSV (95% CI 1.3–15). Clinical differentiation between viral ARTIs was not possible due to common shared presenting signs and symptoms and the high frequency of mixed viral infections. We observed virus-associated outcome differences among children aged < 3 years. Oxygen treatment was associated with RSV (OR 3.6) and inversely correlated with FLU (OR 0.05). Treatment with steroids (OR 3.4) or bronchodilators (OR 3.4) was associated with HRV. Severe respiratory complications were associated with HRV (OR 3.5) and inversely correlated with RSV (OR 0.24).

**Conclusions:**

Respiratory viruses are frequently detected in young children presenting to the hospital emergency department with ARTI and require PCR diagnosis since presenting signs and symptoms are not discriminant for a type of virus. RSV and HRV bear a high burden of morbidity in the pediatric clinical setting.

## Background

Children suffer through multiple episodes of viral acute respiratory tract infection (ARTI) annually and symptoms range from common mild upper respiratory illness to lower respiratory tract infection (LRTI) [1,2]. Large-scale studies during the past decades were unable to provide accurate virus-specific clinical correlations due to the low sensitivity of viral culture and immunofluorescence techniques and inability of these diagnostic methods to detect non-culturable or mixed viral infections [1,3]. The routine implementation of multiplex PCR in recent years has allowed for sensitive and accurate identification of single and mixed viral infections. [4,5].

The burden of individual respiratory viruses remains unclear among different pediatric age-groups in the clinical setting. Recent molecular population-based studies show that viral ARTIs exceed 21% of pediatric emergency department visits during the seasonal influenza winter season and annual hospitalization rates exceed 1.5 per 100 children aged < 3 years [6-8]. These findings suggest a high burden of viral ARTI in young children. The burden of viral respiratory pathogens in children is underestimated since human rhinovirus (HRV) infections were not evaluated in these studies. Recent clinical studies have included HRV molecular diagnostics, but interpretation of virus-associated correlations is hampered by age- and symptom-related enrollment differences [9,10] or by sampling during the 2009 influenza pandemic [11,12].

This study assessed the relative incidence of respiratory virus infections in children presenting to the emergency department of a tertiary hospital with ARTI during two winter seasons and evaluated virus-specific clinical correlations in young children.

## Methods

### Study design and patient populations

This retrospective cohort study included all children aged < 18 years presenting to the emergency department of Leiden University Medical Center (LUMC) during the 2006 and 2007 winter seasons (November-April) with ARTI who were sampled within 48 hours of hospital presentation. The LUMC is a tertiary referral hospital for the south-western part of the Netherlands covering a population of approximately 2 million inhabitants. Clinical diagnosis of ARTI was made by the attending physician. Routine diagnostic specimens were prospectively analysed by respiratory virus multiplex PCR to evaluate the relative incidence of respiratory viruses among different pediatric age-groups (children aged < 3 years and children aged 3–17 years). Structured medical records of children aged < 3 years with single virus infections (RSV, HRV, FLU or Other) were evaluated for baseline characteristics, presenting signs and symptoms and clinical outcomes.

### Respiratory specimens and molecular diagnostics

Qualified medical personnel obtained diagnostic respiratory specimens and a single specimen was evaluated for each child sampled within 48 hours of hospital presentation. Respiratory specimens included nasopharyngeal washes, throat swabs, nasal swabs, sputum or tracheal aspirates. Nucleic acid was extracted by using a MagnaPure LC total nucleic acid kit (Roche Diagnostics, Mannheim, Germany) as described [4]. A fixed amount of equine arteritis virus served as an RNA internal control and phocid herpesvirus was used as a DNA internal control. Respiratory virus multiplex PCR detected respiratory syncytial virus (RSV), human rhinovirus (HRV), influenzavirus (FLU) A/B, parainfluenzavirus (PIV) 1/2/3/4, human metapneumovirus (HMPV), human coronavirus (HCoV) 229E/NL63/OC43, and adenovirus (HAdV) in multiple tubes as described [4]. Primers, probes and amplification methods of the multiplex PCR used in this study are described in reference 4 for ‘laboratory 2’. In a pilot run during the 2007 winter season, we performed additional real-time PCR analysis of HCoV HKU1, *Mycoplasma pneumoniae* and *Chlamydophila pneumoniae* [4]. During this pilot run, we performed additional analysis of human bocavirus (HBoV) by real-time PCR amplification of a 138-bp fragment of the NS1 gene as described by others [13].

### Ethics

The study was conducted in accordance with ethical principles expressed in the World Medical Association’s Declaration of Helsinki. The study procedures complied with legal and regulatory standards and clinical data was obtained by following professional codes of conduct. All necessary precautions were taken to prevent identification of any child included in the study. The Medical Ethics Committee (MEC) of Leiden University Medical Center reviewed the study protocol (C14.128) and final version of the manuscript and confirms that the study is based on clinical data collected in the context of routine clinical practice. For this retrospective analysis of routine clinical data the committee declares that no formal ethical approval and written informed consent is needed.

### Study procedure

#### Viral distribution

Routine diagnostic PCR results from all eligible children were analysed. Distribution of RSV, HRV, FLU and Other single virus infections was depicted by age-groups (young children aged < 3 years and children aged 3–17 years). PIV, HMPV, HAdV and HCoV were aggregated into a distinct group (Other) due to small numbers and to enable more accurate comparative virus-associated correlations.

#### Presenting signs and symptoms

The attending physician recorded signs and symptoms of young children aged < 3 years using structured medical records. Presenting signs and symptoms manifesting within 48 hours of hospital presentation of children with RSV, HRV, FLU or Other single virus infections were evaluated. Virus-associated symptoms were compared and included fever > 38.5°C, cough, rhinitis, pharyngitis, wheezing, crepitations, dyspnea or tachypnea. The clinical presence of chest wall retractions, nasal flaring, moaning or laboured breathing were used to define dyspnea. World Health Organization clinical diagnostic criteria defined tachypnea among children aged < 2 months (≥ 60 breaths per minute, bpm), aged 2 to 12 months (≥ 50 bpm) and aged ≥ 12 months (≥ 40 bpm).

#### Outcomes

The attending physician recorded outcomes of children aged < 3 years with RSV, HRV, FLU and Other single virus infections. We evaluated laboratory and pulmonary imaging findings that were obtained within 96 hours of hospital presentation and adverse outcome manifestations (hospital admission, severe respiratory complications, mortality) that were recorded within ≤ 7 days of hospital presentation. Laboratory findings included C-reactive protein (CRP) and white blood cell count (WBC). We evaluated cut-offs that would indicate absence of serious bacterial infections (CRP < 35 mg/l; WBC < 15 x10^9^/L) [14,15]. We compared bacteriology results that were obtained by PCR (atypical bacteria) or from routine cultures from non-sterile (urine, sputum) and sterile sites (blood, cerebrospinal fluid). Pulmonary imaging findings were assessed for signs of LRTI (radiologic presence of alveolar or peribronchial infiltrates, interstitial opacities, hyperinflation). Supplemental treatments included brochodilators, steroids, oxygen supplementation and antibiotics. Oxygen supplementation was provided during sustained oxygen saturation ≤ 92% or during dyspnea with abnormal PCO2 levels. Adverse outcomes included hospital admission, development of severe respiratory complications and all-cause mortality ≤ 7 days of hospital presentation. Severe respiratory complications were defined as apnea, respiratory intubation and Apparent Life Threatening Events (ALTE). ALTE was defined as an acute change in an infant’s breathing behavior perceived as possibly life threatening by the child’s caretaker.

### Statistical analysis

Statistical analyses were performed using SPSS software version 20.0 (SPSS, Chicago, IL). Continuous variables were presented as mean or median (with range) and categorical variables as frequencies (with percentages). Mann–Whitney U-test and Kruskal–Wallis test were used as non-parametric tests to compare age (months) between 2 or more groups, because of non-normally distributed data. Chi square was performed to compare categorical variables between 2 groups. Logistic regression analysis compared categorical variables between virus groups including presenting signs and symptoms, diagnostic, treatment and outcome findings. Multivariate analyses adjusted for age, gender and relevant history or chronic underlying disorders (stepwise). A 2-sided value of *P* < 0.05 was considered significant.

## Results

### Patient enrollment and viral etiology

**Figure 1:**
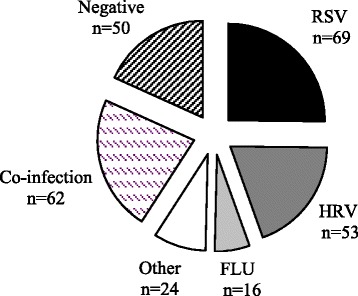
**Viral etiologies of 274 children with ARTI presenting to the emergency department of a tertiary referral hospital.**

During two winter seasons, we enrolled 274 children presenting to the emergency department with ARTI and sampled within 48 hours of hospital presentation. The majority of children were aged < 3 years (236/274, 86%) compared to older children aged 3–17 years (38/274, 14%). Multiplex PCR detected respiratory viruses in 82% (224/274) of all children and included single virus infections (162 of 224, 72%) and mixed viral infections (62 of 224, 28%) (Figure 1). Single virus infections were caused by RSV (69 of 224, 31%), HRV (53 of 224, 24%), FLU (16 of 224, 7%) and Other (24 of 224, 11%). In a pilot run among a total 131 children during the 2007 winter season, PCR yielded HBoV (7%) and HCoV HKU1 (1%), but no *Mycoplasma pneumoniae* and *Chlamydophila pneumoniae*. HBoV- and HCoV HKU1-associated clinical findings were not evaluated due to a rare occurrence of single virus infections (n = 4 HBoV; n = 0 HCoV HKU1) and due to incomplete data (single season).

### Respiratory virus distribution

**Figure 2:**
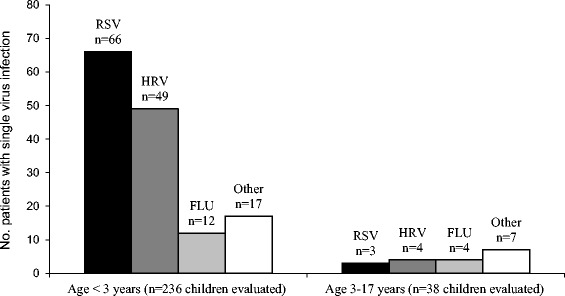
**Distribution of single respiratory virus infections among different pediatric age groups.**

The distribution of single virus infections among different age groups was evaluated in an attempt to establish virus-associated clinical correlations. Mixed viral infections were not analyzed due to the small sample size and the difficulty to establish clinical relevance. Single virus infections were common among young children aged < 3 years (144 of 236, 61%) compared to older children (18 of 38, 47%) but these proportional differences were not statistically significant (Figure 2). RSV and HRV were more common among children aged < 3 years compared to older children, but proportional differences were only significant for RSV (28% vs 7.9%; 95% CI 1.3–15). In contrast, young children were less common infected with Other single viruses (including PIV, HMPV, HAdV or HCoV) compared to older children (7.2% vs 18%; 95% CI 0.13–0.90). FLU single virus infections were uncommon in both age groups with a similar low prevalence (range, 5 to 11%).

### Virus-specific clinical correlations

#### Baseline characteristics

Evaluation of baseline characteristics was limited to children aged < 3 years with single virus infections (RSV, HRV, FLU, Other ) with clinical data available. We included demographics (sex, age) and relevant history encompassing prematurity at birth (gestational age < 37 weeks), bronchial hyperreactivity (bronchoconstriction in response to stimuli) and underlying pulmonary, cardiovascular and immunodeficiency disorders. Base-line characteristics were equally distributed among virus groups, except for a male predominance (Table 1) among children with HRV vs FLU (73% vs 36%; 95% CI 1.2–19). Proportional differences at baseline underscore the need for statistical adjustment for possible confounders during comparative analyses.

**Table 1:** **Baseline characteristics of children aged < 3 years with single respiratory virus infections**

	**Total**	**RSV**	**HRV**	**FLU**	**Other**
	**N (%)**	**N (%)**	**N (%)**	**N (%)**	**N (%)**
**Children <3 y with single virus ARTI**					
Total included	144	66	49	12	17
Analysis of signs and symptoms (data available)	138	64	46	11	17
Analysis of clinical outcomes (data available)	123	53	44	11	15
**Demographics** (analysis of clinical outcomes)					
Male	76 (62)	30 (57)	32 (73)^$^	4 (36)^$^	10 (67)
Mean age, months [range]	8 [0–35]	7 [0–35]	7 [0–24]	10 [1–26]	11 [0–33]
Median age, months [range]	4 [0–35]	3 [0–35]	4 [0–24]	9 [1–26]	8 [0–33]
**Clinical history** (analysis of clinical outcomes)					
Prematurity at birth	11 (9)	6 (11)	3 (7)	1 (9)	1 (7)
History of BHR	12 (9)	5 (9)	5 (11)	1 (9)	1 (7)
Chronic pulmonary disorder	2 (2)	0	2 (5)	0	0
Chronic cardiovascular disorder	12 (10)	3 (6)	6 (14)	1 (9)	2 (13)
Chronic immunodeficiency disorder	5 (4)	0	2 (5)	1 (9)	2 (13)

Baseline characteristics are equally distributed, except for a male predominance with HRV vs FLU (95%CI 1.2-19)^$^.

#### Signs and symptoms

**Figure 3:**
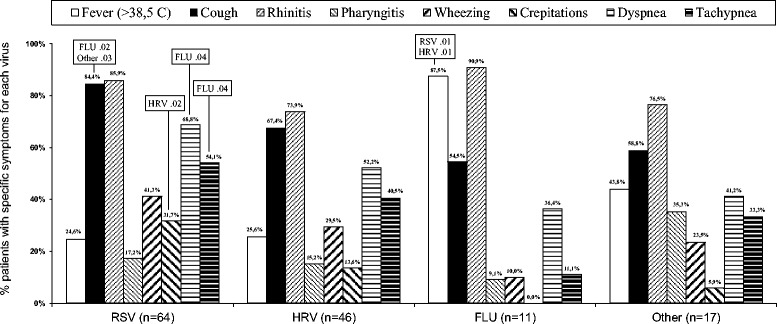
**Signs and symptoms of 138 children aged < 3 years presenting to the emergency department of a tertiary referral hospital with single virus ARTI.** The proportions of presenting signs and symptoms were compared between virus groups using logistic regression analysis and multivariate adjustment for demographics and relevant clinical history (stepwise). Significant proportional differences (P < 0.05) between virus groups are depicted and include fever (FLU vs RSV, P = 0.01; FLU vs HRV, P = 0.01) and other findings (cough, crepitations, dyspnea and tachypnea).

Presenting signs and symptoms were compared among 138 children aged < 3 years with single virus infections and clinical data available (Table 1, Figure 3). Six children were previously excluded from comparative analyses due to incomplete documentation. Children aged 3–17 years were not evaluated due to small numbers and age-related confounding differences. Multivariate analyses adjusted for possible confounders including age and sex, and stepwise for relevant underlying disorders. Significant virus-associated presenting signs and symptoms (Figure 3) included fever (FLU vs RSV, *P =* 0.01; FLU vs HRV, *P =* 0.01), cough (RSV vs FLU, *P =* 0.02; RSV vs Other, *P =* 0.03), crepitations (RSV vs HRV, *P =* 0.02), dyspnea and tachypnea (RSV vs FLU, *P =* 0.04).

#### Clinical outcomes

Clinical outcomes were compared among 123 children aged < 3 years with single virus infections and clinical data available (Table 1, Table 2). A total of 21 children were previously excluded from comparative analyses due to incomplete documentation or hospital transfer without follow-up data. Excluded cases were equally distributed among virus groups (Table 1). High levels of CRP (≥ 35 mg/l) or WBC (≥ 15 x10^9^/L) and antibiotic treatment were equally distributed among virus groups and suggested no differences in potential serious bacterial infections. Bacterial cultures and PCRs were often negative and were equally distributed among individual virus infections. This supports the assumption that there were no differences in potential serious bacterial infections among virus groups. Hospital admission (~80%) was equally distributed and fortunately there was no mortality. Overall and individual comparisons were made among children with RSV, HRV, FLU and Other single virus infections (Table 2) following multivariate adjustment for possible confounders.

**Table 2:** **Outcomes associated with single respiratory virus infections in children aged < 3 years**

	**Single virus ARTI**					**Statistical analysis**		
	**Total**	**RSV**	**HRV**	**FLU**	**Other**	**vs**	**Multivariate** ^**$**^	**P**
	**N (%)**	**N (%)**	**N (%)**	**N (%)**	**N (%)**		**OR (95%CI)**	**value**
**Diagnostics**								
Chemistry/hematology								
CRP ≥ 35 mg/L	28/81 (35)	11/32 (34)	11/29 (38)	2/9 (22)	4/11 (36)	…	…	NS
WBC ≥ 15 x109/L	27/80 (34)	11/32 (34)	11/28 (39)	2/9 (22)	3/11 (27)	…	…	NS
Bacteriology								NS
PCR atypical bacteria	0/10 (0)	0/2 (0)	0/4 (0)	0/1 (0)	0/3 (0)	…	…	NS
Culture, sterile site	2/45 (4)	0/15 (0)	1/17 (6)	0/7 (0)	1/6 (17)	…	…	NS
Culture, non-sterile site	1/19 (5)	0/7 (0)	0/8 (0)	0/1 (0)	1/3 (33)	…	…	NS
Pulmonary imaging								
LRTI^#^	31/63 (37)	15/24 (63)	10/25 (40)	3/6 (33)	3/8 (38)	…	…	NS
**Treatment**								
Antibiotics	50/122 (41)	24/53 (45)	17/43 (40)	3/11 (27)	6/15 (40)	…	…	NS
Bronchodilators	31/113 (27)	12/48 (25)	15/39 (38)	2/11 (18)	2/15 (13)	HRV vs Other	7.0 (1.2-42)	.03
						*HRV overall*	*3.0 (1.2-7.8)*	.02
Steroids	26/117 (22)	8/52 (15)	13/39 (33)	2/11 (18)	3/15 (20)	HRV vs RSV	3.5 (1.2-11)	.03
						*HRV overall*	3.4 (1.2-9.3)	.02
Oxygen therapy	67/118 (57)	38/52 (73)	22/41 (54)	1/10 (10)	6/15 (40)	RSV vs FLU	26 (3.0-225)	< .01
						RSV vs Other	4.2 (1.3-14)	.02
						HRV vs FLU	11 (1.2-97)	.03
						*RSV overall*	*3.6 (1.6-8.0)*	< .01
						*FLU overall*	.05 (.01-.40)	< .01
**Adverse outcome**								
Hospital admission	99/123 (80)	46/53 (87)	33/44 (75)	8/11 (73)	12/15 (80)	…	…	NS
Severe respiratory complication	15/123 (12)	3/53 (6)	9/44 (20)	1/11 (9)	2/15 (13)	HRV vs RSV	5.0 (1.2-21)	.03
						*HRV overall*	*3.5 (1.0-11)*	.04
						*RSV overall*	*.24 (.06-.92)*	.04
Apnea	5/123 (4)	1/53 (2)	4/44 (9)	0/11 (0)	0/15 (0)	…	…	NS
Intubation	7/123 (6)	1/53 (2)	5/44 (11)	0/11 (0)	1/15 (7)	…	…	NS
ALTE	6/123 (5)	1/53 (2)	3/44 (7)	1/11 (9)	1/15 (7)	…	…	NS
Mortality ≤ 7 days	0/123 (0)	0/53 (0)	0/44 (0)	0/11 (0)	0/15 (0)	…	…	NS

ARTI, Acute Respiratory Tract Infection, CRP, C-reactive protein; WBC, White Blood Cell count; LRTI, lower respiratory tract infection; ALTE, Apparent Life Threatening Events; NS, not significant.

^#^LRTI was defined as radiologic presence of alveolar infiltrates, interstitial opacities, peribronchial infiltrates or hyperinflation.

^$^Multivariate correction for sex, age and underlying disease (forward stepwise adjustment) using logistic regression analysis.

#### RSV

Overall, RSV infection was associated with supplemental oxygen requirement (OR 3.6) and inversely correlated with severe respiratory complications (OR 0.24). Individual comparisons revealed that RSV was associated with supplemental oxygen requirement compared to FLU (OR 26) and Other (OR 4.2).

#### HRV

Overall, HRV infection was associated with bronchodilator therapy (OR 3.0), steroid treatment (OR 3.4) and severe respiratory complications (OR 3.5). Individual comparisons revealed that HRV was associated with bronchodilator therapy compared to Other (OR 7.0), steroid treatment compared to RSV (OR 3.5), supplemental oxygen requirement compared to FLU (OR 11) and severe respiratory complications (apnea, respiratory intubation or ALTE) compared to RSV (OR 5.0). HRV subtyping was not performed and therefore specific subtypes could not be associated with clinical outcome.

#### FLU

Overall, Flu infection was inversely correlated with supplemental oxygen requirement (OR 0.05). Individual comparisons revealed no FLU specific outcome associations.

## Discussion

This study confirmed a frequent viral etiology among 82% (224 of 274) of children aged < 18 years presenting to the emergency department of a tertiary hospital with ARTI. These findings add to a growing body of literature on the epidemiology and virus-associated clinical features in the clinical setting [6-12].

The high detection rate of respiratory viruses using multiplex PCR was similar to previously published rates exceeding 80% in the pediatric clinical setting [10,11]. Much lower detection rates (range 58% - 67%) in a few other studies are likely due to age- and symptom-related enrollment differences or due to sampling during non-winter seasons [9,12]. A high rate of mixed viral infections in this study (23%) is similar to findings in other studies (range 14% - 30%) [9-11].

Children presenting to the hospital emergency department with ARTI and sampled within 48 hours were predominantly aged < 3 years (236 of 274, 86%). HRV and RSV single virus infections were common among children aged < 3 years, but proportional differences compared to older children were only significant for RSV (95% CI 1.3–15). Previous studies report a similar predominance of RSV among young children in the clinical setting [7,8,10] but reports on HRV age-distribution are mixed [10,16]. FLU cases were often young children (n = 12) compared to older children (n = 4) but the small numbers and the lack of a population-based design restrict firm epidemiologic conclusions.

Viral ARTI signs and symptoms are widely presumed to be aspecific but confirmation of this assumption is lacking. This study confirmed that viral ARTI presenting signs and symptoms are aspecific by comparative statistical analysis among children aged < 3 years with single virus infections. Fever was often associated with influenza (FLU vs RSV, *P =* 0.01; FLU vs HRV, *P =* 0.01) and cough was often associated with RSV (RSV vs FLU, *P =* 0.02; RSV vs Other, *P =* 0.03). Unfortunately, these and other findings were insufficient to differentiate between individual viral ARTIs due to common shared signs and symptoms among viruses and the high frequency of mixed viral infections [9]. A febrile disease with ARTI symptoms was observed among ≥ 25% of total viruses infections, therefore influenza-like illness required PCR confirmation to establish FLU diagnosis [17,18].

Virus-specific comparative outcome analysis among children aged < 3 years with single virus infections unveiled clinical outcome similarities (Table 2). Laboratory infection parameters (CRP, WBC), pulmonary imaging LRTI findings and antibiotic use were similar among virus groups. The findings contrast with a previous study which reported high CRP levels, elevated leucocyte counts and frequent antibiotic use during HRV [10]. In this study, children with RSV often received antibiotics (45%). Previous studies show that rapid confirmation of RSV can limit antibiotic use [19,20], but antibiotic stewardship guided by respiratory virus PCR results may be difficult to implement [21].

Relevant virus-specific outcome differences include supplemental oxygen treatment requirement (RSV), steroid and bronchodilator treatment (HRV) and development of severe respiratory complications (HRV). Supplemental treatments with corticosteroids and bronchodilators were remarkably associated with HRV. There is insufficient evidence on the benefit of corticosteroids or bronchodilators during viral LRTI and most guidelines do not recommend the routine use for cases of RSV LRTI [22]. However, corticosteroids may improve HRV-induced wheezing and bronchodilators may be effective for individual children experiencing LRTI with underlying reactive-airway disease. This may explain why corticosteroids and bronchodilators were more commonly provided to children with HRV. Young children presenting to the hospital emergency department with the combined symptoms of cough, pulmonary crepitation and oxygen-dependent viral LRTI were more likely to be infected with RSV than with other viruses as reported by others [12,23]. With the knowledge that RSV is more often associated with oxygen-dependent viral LRTI, it would seem counter-intuitive that HRV (and not RSV) is associated with more severe respiratory complications (Table 2). HRV often causes mild symptoms, but the findings of this and other studies suggest that the occurrence of severe HRV respiratory complications may be underestimated [24,25]. In this study, FLU infection was inversely correlated with supplemental oxygen treatment compared to other infections as described by others [26]. We emphasize that the low number of FLU cases does not allow for any firm conclusions on this matter. Previous studies suggest that FLU manifestations may be relatively mild among young children presenting to the hospital during seasonal influenza, but rare life-threatening events and severe cases of concomitant bacterial pneumonia do occur [12,27].

### Limitations

Children were evaluated at a tertiary care setting and this possibly limits the validity of virus-specific clinical correlations in other settings. The retrospective design of the study and evaluation of the patients by different attending physicians may have introduced a reporting bias. The inclusion of children sampled within 48 hours of hospital presentation may have introduced a selection bias towards younger patients due to a more cautious clinical and diagnostic approach of parents and paediatricians in that age group [28]. This could explain why the majority of children included in this study were aged < 3 years (236/274, 86%).

The respiratory specimens were of different types (including nasopharyngeal washes, throat swabs, nasal swabs, sputum or tracheal aspirates) which could have bearing on the sensitivity of the PCR for the various viruses and thus their ‘relative frequency’. The number of children with viral ARTI is an underestimation since our multiplex PCR did not detect HBoV, HCoV HKU1, human influenza C virus and enteroviruses that may cause viral respiratory infections. Molecular differentiation of HRV and enteroviruses is difficult and the HRV assay used in this study cross-reacts with a few enteroviruses that are associated with respiratory infections. This variation in HRV types may explain severe ‘HRV’ infections and emphasizes that future studies should elucidate the specific role of HRV and enteroviruses using molecular subtyping.

In this study, multiple comparisons were performed which can lead to more type I errors (more false positives) since 5% of the comparisons have uncorrected P values < 0.05. Statistical adjustment of confidence intervals (eg Bonferroni corrections) can be applied to reduce incorrect rejection of true null hypotheses and to lower type I errors, but these corrections can increase the type II error (more false negatives) and lead to interpretation errors [29]. In this observational study, no adjustments were made for multiple comparisons in an attempt to find novel virus-specific clinical correlations. This approach is intended for hypothesis generation and not for hypothesis testing. Future prospective studies using improved standardized study protocols are therefore awaited to confirm virus-associated clinical findings and for hypothesis testing.

## Conclusions

This study confirmed that a viral agent is frequently found in young children with ARTI presenting to the pediatric emergency department of a tertiary referral hospital. Molecular diagnostics are required to confirm respiratory virus infections since presenting symptoms could not discriminate between the viruses. RSV and HRV infections bear the highest burden of morbidity in the pediatric clinical setting.

## Abbreviations

ARTI: Acute respiratory tract infection
BHR: Bronchial hyperresponsiveness
Bpm: Breaths per minute
CRP: C-reactive protein
FLU: Influenzavirus
HAdV: Human adenovirus
HBoV: Human bocavirus
HCoV: Human coronavirus
HMPV: Human metapneumovirus
HRV: Human rhinovirus
LRTI: Lower respiratory tract infection
OR: Odds ratio
PCR: Polymerase chain reaction
PIV: Parainfluenzavirus
RSV: Respiratory syncytial virus
WBC: White blood count
